# Lymphatic Vessels of the Tongue

**Published:** 1849-07

**Authors:** 


*Lymphatic Vessels of the Tongue.*—M. Sappey has addressed a communication to the Academy of Sciences, on the lymphatic vessels of the tongue. According to this observer, vessels of this description exist in great number beneath the epidermis of the tongue. If that organ be carefully injected, they are found to be thickly dispersed over the greater part of its mucous membrane, so much so, that the tongue might be looked upon as an organ contained in a sheath of lymphatics. Next to the glands, on which absorbents are distributed in the greatest abundance, there is perhaps no free surface on which lymph-channels are distributed in so great numbers.

From the researches which he has made, M. Sappey arrives at the following conclusions: 1. That there exists on the surface of the tongue a network of two kinds; one of lymphatics, another of veins; but the former is always the more superficial, and may be injected independently of the latter. 2. The network observable on the mucous membrane lining the nasal fossæ is exclusively made up by venous ramifications.

M. Borraney, in his "Anatomical Atlas," has well figured the network of vessels described on the tongue.

